# Clinical-Functional Vulnerability Index-20 (IVCF-20): rapid recognition of frail older adults

**DOI:** 10.1590/S1518-8787.2016050006963

**Published:** 2016-11-24

**Authors:** Edgar Nunes de Moraes, Juliana Alves do Carmo, Flávia Lanna de Moraes, Raquel Souza Azevedo, Carla Jorge Machado, Dalia Elena Romero Montilla

**Affiliations:** I Núcleo de Geriatria e Gerontologia. Departamento de Clínica Médica. Faculdade de Medicina. Universidade Federal de Minas Gerais. Belo Horizonte, MG, Brasil; II Programa de Pós-Graduação em Promoção da Saúde e Prevenção da Violência. Universidade Federal de Minas Gerais. Belo Horizonte, MG, Brasil; III Núcleo de Geriatria e Gerontologia. Secretaria Municipal da Saúde. Prefeitura de Belo Horizonte. Belo Horizonte, MG, Brasil; IV Núcleo de Geriatria e Gerontologia. Hospital das Clínicas. Universidade Federal de Minas Gerais. Empresa Brasileira de Serviços Hospitalares. Belo Horizonte, MG, Brasil; V Núcleo de Geriatria e Gerontologia. Departamento de Medicina Preventiva e Social. Faculdade de Medicina. Universidade Federal de Minas Gerais. Belo Horizonte, MG, Brasil; VI Laboratório de Informação em Saúde. Instituto de Comunicação e Informação Científica e Tecnológica em Saúde. Fundação Oswaldo Cruz. Rio de Janeiro, RJ, Brasil

**Keywords:** Frail Elderly, Geriatric Assessment, Triage, methods, Primary Health Care, Health Vulnerability

## Abstract

**OBJECTIVE:**

To evaluate the adequacy of the Clinical-Functional Vulnerability Index-20, a rapid triage instrument to test vulnerability in Brazilian older adults, for the use in primary health care.

**METHODS:**

The study included convenience sample of 397 patients aged older than or equal to 60 years attended at *Centro de Referência para o Idoso* (Reference Center for Older Adults) and of 52 older adults the same age attended at the community. The results of the questionnaire, consisting of 20 questions, were compared with those of the Comprehensive Geriatric Assessment, considered a reference for identifying frail older adults. Spearman’s correlation was evaluated in the Clinical-Functional Vulnerability Index-20 with the Comprehensive Geriatric Assessment; the validity was verified by the area under the ROC curve; reliability was estimated by the percentage of agreement among evaluators and by the kappa coefficient, both with quadratic weighted. The cut-off point was obtained based on the higher accuracy criterion. Cronbach’s alpha, a measure of internal consistency, was estimated.

**RESULTS:**

The Spearman’s correlation coefficient was high and positive for both groups (0.792 for older adults attended at the Reference Center and 0.305 for older adults from the community [p < 0.001]). The area under the ROC curve for older adults attended at the Reference Center was substantial (0.903). The cut-off point obtained was six, and older adults with scores in Clinical-Functional Vulnerability Index-20 above that value had strong possibility of being frail. For older adults from the community, the quadratic weighted agreement among evaluators was 99.5%, and the global quadratic weighted kappa coefficient was 0.94. Cronbach’s alpha was high for older adults attended at the Reference Center (0.861) and those attended at the community (0.740).

**CONCLUSIONS:**

The Clinical-Functional Vulnerability Index-20 questionnaire, in the sample examined, turned out to be positively correlated with the Comprehensive Geriatric Assessment, in addition to the results indicating a high degree of validity and reliability. Thus, the Clinical-Functional Vulnerability Index-20 proves to be viable as a triage instrument in the primary health care that identifies frail older adults (older adults at risk of weakening and frail older adults).

## RESUMO

**OBJETIVO:**

Avaliar a adequação do Índice de Vulnerabilidade Clínico-Funcional-20 , instrumento de triagem rápida de vulnerabilidade em idosos brasileiros, para utilização pela atenção básica. O estudo incluiu amostra de conveniência de 397 pacientes com idade maior ou igual a 60 anos atendidos em um Centro de Referência para o Idoso e de 52 idosos da mesma idade atendidos na comunidade. Os resultados do questionário, constituído por 20 perguntas, foram comparados com aqueles da Avaliação Geriátrica Ampla, considerada referência para identificação do idoso frágil. Foi avaliada a correlação de Spearman do Índice de Vulnerabilidade Clínico-Funcional-20 com a Avaliação Geriátrica Ampla; a validade foi verificada pela área sob a curva ROC; a confiabilidade foi estimada pelo percentual de concordância entre avaliadores e coeficiente kappa, ambos com ponderação quadrática. Obteve-se ponto de corte com base no critério de maior acurácia. O alfa de Cronbach, medida de consistência interna, foi calculado. O coeficiente de correlação de Spearman foi elevado e positivo em ambos os grupos (0,792 para idosos atendidos no Centro de Referência para o Idoso e 0,305 para idosos da comunidade [p < 0,001]). A área sob a curva ROC para idosos atendidos no Centro de Referência para o Idoso foi substancial (0,903). O ponto de corte obtido foi seis e idosos com pontuação no Índice de Vulnerabilidade Clínico-Funcional-20 acima desse valor tinham forte possibilidade de serem frágeis. Para idosos da comunidade, a concordância ponderada quadrática entre avaliadores foi 99,5% e o coeficiente kappa ponderado quadrático global, 0,94. O alfa de Cronbach foi elevado para idosos atendidos no Centro de Referência para o Idoso (0,861) e da comunidade (0,740).

**CONCLUSÕES:**

O questionário do Índice de Vulnerabilidade Clínico-Funcional-20, na amostra analisada, mostrou ser positivamente correlacionado com a Avaliação Geriátrica Ampla, além de os resultados indicarem alto grau de validade e confiabilidade. Assim, o Índice de Vulnerabilidade Clínico-Funcional-20 se mostra viável como instrumento de rastreio na atenção básica que identifica o idoso com fragilidade (idoso em risco de fragilização e idoso frágil).

## INTRODUCTION

Aging is closely associated with the weakening process. However, age, by itself, is an inappropriate fragility predictor, since the aging process follows a heterogeneous pattern. The chronological age is just a poor approximation of the biological age^4^. Thus, heterogeneity among older adults is important and progressive throughout the aging process. Similarly, aging without any chronic disease is infrequent^20^. Hence, knowing only the age of the individuals and the number of chronic diseases does not aggregate possibilities for a greater understanding of the situation of health and capacity of older adults. Thus, older adults’ health can be understood as the individual capacity of satisfying biopsychosocial needs, regardless of age or the presence of diseases.

The term fragility is commonly used to represent the degree of vulnerability of older adults to adverse outcomes, such as functional decline, falls, hospitalization, institutionalization, and death. However, the term presents several definitions, depending on the proportion used for reference, hindering its standardization and implementation in the clinical practice and in the comparison between different studies^5^ ^,^ ^17^. Moraes et al. ^11^, in a recent study, consider as multidimensional fragility the decrease in the homeostatic reserve or in the capacity of adaptation to biopsychosocial assaults and, consequently, the increased vulnerability to functional decline and its consequences.

The Comprehensive Geriatric Assessment (CGA) is the primary tool used to identify frail older adults and must be applied by a geriatric/gerontological specialized team, in which several scales or instruments are used^10^. Its average length ranges from 60 to 90 minutes^12^. Thus, we consider CGA as a diagnostic procedure of high cost that needs to be well prescribed. Therefore, it is essential to use rapid triage instruments, applied by any health professional, such as communitarian health agents or nursing technicians, able to recognize older adults at risk. Although several instruments for rapid triage of vulnerability in older adults are described in the literature, those that could be used in primary health care still have an incipient validation for practical use^6^. Triage instruments available for older adults lack the accuracy required for identifying frail older adults^2^. We also found no studies that assess the insertion of these instruments in integral management of older adults in the long term, both on the primary health care and secondary care^2^.

In Brazil, primary health care professionals tend to consider older adults as frail based on their general appearance, or when such individuals feature multiple diseases or comorbidities. For these professionals, the proper identification of frail older adults or those at risk of weakening needs to be simple and fast. Some studies tested the effectiveness of some instruments in the identification of fragility in older adults on primary health care^1^ ^,^ ^13^ ^,^ ^15^ ^,^ ^16^, but none of these instruments was specifically designed to identify frail older adults, according to the conception of higher vulnerability to functional decline^10^ ^-^ ^12^. In addition, studies in developing countries focused on finding an instrument for such identification are scarce.

Thus, the objective of this study was to assess the adequacy of the Clinical-Functional Vulnerability Index-20 (IVCF-20) as an instrument for screening fragility to be used by health professionals in Brazil. Therefore, CGA was used as reference standard.

CGA allows a global and comprehensive diagnostic process, involving patients and their family, to verify the health of older adults as a whole. It consists in the search of information pertaining to several aspects: global functionality, functional systems (cognition, mood, mobility, communication), physiological systems, use of medication, prior medical history, and contextual factors (socio-family, environmental, and caregiver’s evaluation). It allows classifying older adults in one of 10 clinical-functional strata^11^. An individual in the ≥ 4 clinical-functional stratum is considered frail (Chart). However, the use of CGA in the context of primary health care is unfeasible, showing a poor cost-benefit ratio in public health. Thus, it is important to define who is the older person being subjected to this evaluation^12^, and in the same way are objective, simple, and fast-application multidimensional triage instruments.

**Chart:** Clinical-Functional classification of older adults, according to Moraes et al.11

Group	Stratum	Clinical-Functional classification
Robust older adults	Stratum 1	They are at their maximum degree of vitality. They present independence for all advanced, instrumental, or basic activities of daily living, and absence of disease or risk factors except age itself.
	Stratum 2	They are independent for all activities of daily living, but present health conditions of low clinical complexity, such as uncomplicated hypertension or presence of risk factors such as smoking, dyslipidemia, osteopenia, among others.
	Stratum 3	They are independent for all activities of daily living, but present well established chronic-degenerative diseases of higher complexity, such as complicated hypertension, diabetes mellitus, history of transient ischemic attack, cerebrovascular accident without sequelae, chronic kidney disease, heart failure, chronic obstructive pulmonary disease, osteoarthritis, coronary artery disease, peripheral artery disease, osteoporosis, atrial fibrillation, depression, among others. In these older adults, such diseases are not associated with functional impairment and are presented in isolation. In this group, are also included older adults who feature one or two criteria of the “fragility phenotype”, according to Fried and Ferrucci^4^.
Older adults at risk of frail	Stratum 4	They are independent for all activities of daily living, but present predictive conditions of adverse outcomes represented by the higher risk of functional decline established, institutionalization, or death: presence of sarcopenia markers, mild cognitive impairment, or multiple comorbidities (polypathology, polypharmacy, or recent hospitalization). In this group, are included older adults who have three or more criteria of the “fragility phenotype”, according to Fried and Ferrucci^4^.
	Stratum 5	They present predictive conditions of adverse outcomes (as in Stratum 4), but have functional decline in activities of daily living, associated with leisure, work, or social interaction. These older adults are still independent for instrumental and basic activities of daily living.
Frail older adults	Stratum 6	They present partial functional decline in instrumental activities of daily living and are independent for the basic activities.
	Stratum 7	They present functional decline in all instrumental activities of daily living, but are still independent for the basic activities.
	Stratum 8	They present complete dependency in instrumental activities of daily living associated with the semi-dependence in the basic activities: impairment of one of the functions influenced by culture and learning – bathing, dressing up, and using the toilet.
	Stratum 9	They present complete dependency in instrumental activities of daily living associated with the incomplete dependence in the basic activities: impairment of one of the simple vegetative functions (transfer and continence), in addition to clearly being dependent for bathing, dressing up, and using the toilet. The isolated presence of urinary incontinence should not be considered.
	Stratum 10	They are at their maximum degree of fragility and, consequently, have the most functional dependency, needing help even to feed themselves.

With this purpose, the IVCF-20 was built in an interdisciplinary way, with the participation of several professionals from a geriatric/gerontological team specialized in the care of older adults. Furthermore, communitarian health agents, nursing auxiliaries and technicians, medical-nurse, *Núcleo de Apoio à Saúde da Família* teams (NASF – Support Center for Family Health), and primary health care managers. The topic was discussed by primary health care professionals from the Southeast, Midwest, North, and South of Brazil, in meetings and workshops at the Brazilian Ministry of Health, with the participation of researchers in the area.

## METHODS

This is a cross-sectional study, with convenience sample, that compared the results obtained by the use of the IVCF-20 questionnaire^a^ with the results verified by the use of CGA.

The IVCF-20 is a questionnaire that covers multidimensional aspects of the older adult’s health condition and has 20 questions divided into eight sections: age (one question), health self-perception (one question), functional disabilities (four questions), cognition (three questions), mood (two questions), mobility (six questions), communication (two questions), and multiple comorbidities (one question). Each section has a specific score that compose a maximum amount of 40 points. The higher the value obtained, the higher the risk of clinical-functional vulnerability of the older adult.

The data of this study were obtained from 449 patients attended in 2014 at the *Centro de Referência do Idoso* (CRI – Reference Center for Older Adults) of the Teaching Hospital of Universidade Federal de Minas Gerais – Instituto Jenny de Andrade Faria de Atenção ao Idoso (Jenny de Andrade Faria Institute of Older Adults Health Care). Of the sample, 397 older adults were evaluated at the institute and submitted to IVCF-20 and CGA. The IVCF-20 was applied by the nursing team before the geriatric care. The CGA was applied by a geriatric/gerontological specialized team. The other 52 patients were evaluated by the CRI team in their community, i.e., in their respective health center.

All patients were evaluated by both questionnaires: IVCF-20 and CGA. However, patients evaluated in their respective health center were subjected to the IVCF-20 questionnaire twice, by two health professionals (here identified as A and B) who did not know of the result obtained by each other.

The training of health professionals for applying the questionnaire was done by the authors of this study, at the CRI and at the health center.

The Statistical Package for Social Sciences – Statistics for Windows (SPSS), version 19.0, was used to build the database. This study was approved by the Research Ethics Committee of Universidade Federal de Minas Gerais (CAAE 35321914.0.0000.5149).

### Data Analysis

The data were analyzed using the Stata^®^ software for MAC, version 12.0. We obtained the absolute and relative frequency of each IVCF-20 question (categorical variables) as well as average, median, and standard deviation for continuous variables. The older adults evaluated at the institute (CRI) and those evaluated at community health centers were separately described.

To verify the correlation between CGA and IVCF-20, we estimated Spearman’s correlation coefficient between these two indicators. To evaluate the validity of the IVCF-20 questionnaire compared with CGA, we obtained the area under the Receiver Operating Characteristic curve (ROC). The area under the ROC curve (AUC) is a summary measure of the performance of a test in relation to the gold standard, that is, the ability in the new test to discriminate the sick individual from the healthy one^8^. The interpretation of this measure is the probability of the individual with a disease or under a condition to have a result to the diagnostic test of greater magnitude than that of a healthy person or an individual without the condition. The higher this probability, the closer the AUC-ROC is to 1. If it is less than 0.50, the probability of discriminating correctly is the same of a test that classifies at random.

The use of the ROC curve enables to establish the best empirical cut-off point, since the programs that implement the ROC curve also estimate the number of correctly classified individuals, considered here as the cut-off point that maximized sensitivity and specificity jointly. After obtaining this cut-off point, variations in accuracy resulting from the use of IVCF-20 instead of the *Avaliação Multidimensional do Idoso* (AMI – Multidimensional Evaluation of Older Adults) were evaluated, with the description of the sensitivity, specificity, and positive and negative predictive values.

Reliability was assessed by correlation between evaluators for older adults evaluated at the community, and by the internal consistency, for the same older adults and those evaluated at CRI. To assess the agreement among evaluators, we used the kappa coefficient for each item and the quadratic weighted coefficient kappa for the global IVCF-20^7^. The classifications adopted for the coefficient were: values less than 0 (zero) indicated the absence of agreement; from 0 to 0.20, slight agreement; from 0.21 to 0.40, considerable agreement; from 0.41 to 0.60, moderate agreement; from 0.61 to 0.80, substantial agreement; from 0.81 to 1.00, excellent agreement^7^. On the other hand, internal consistency was evaluated by the correlation between responses of IVCF-20, by the analysis of the responses of older adults, or of relative homogeneity between a set of multiple items in the IVCF-20. This internal consistency was made explicit by a Cronbach’s alpha coefficient that varies, mainly, from zero to +1. The higher the internal simultaneous association between variables, the higher the reliability, which is measured by the Cronbach’s alpha. DeVellis^3^ believes that values from 0.70 to 0.80 are reputable; from 0.81 to 0.90, very good; above 0.90, the researcher should consider shortening the scale, since this high value may be due to redundant questions^18^ ^,^ ^19^.

The significance level considered was 5%. We obtained a 95% confidence interval for AUC-ROC.

## RESULTS

### Overview of the Sample

Table 1 presents the frequencies (absolute and relative) of the items of the IVCF-20 in the group evaluated at the CRI and the group of older adults from the community.

**Table 1:** Frequencies (absolute and percentage values) of clinical-functional vulnerability indicators.

Clinical-Functional Vulnerability Indicators	CRI		Health Center	
	
	n	%	n	%
Age in years				
60-74	178	44.8	39	75.0
75-84	162	40.8	11	21.2
≥ 85	57	14.4	2	3.9
Regular or bad self-perception of health	236	59.5	10	19.2
Functional disabilities				
Disability in at least one instrumental ADL	154	38.8	1	1.9
Stopped bathing alone for physical condition – basic ADL	58	14.6	1	1.9
Cognition				
A relative or friend mentioned forgetfulness of the patient	245	61.7	5	9.6
Worsening of forgetfulness in the last months^b^	176	71.8^a^	2	40.0^a^
Forgetfulness prevents performing some daily activity	129	52.7^a^	0	0^a^
Mood				
Dismay, sadness, or hopelessness in the last month	204	51.4	2	3.9
Loss of interest or pleasure, in the last month, in previously enjoyable activities	126	31.7	2	3.9
Mobility: reach, graspingness, and pincer grip				
Inability to raise the arm above shoulder level	35	8.8	0	0
Inability to handle or hold small objects	26	6.6	0	0
Aerobic and muscle capacity				
Unintentional weight loss^c^ or BMI < 22 kg/m^2^ or calf circumference < 31 cm or time during the gait speed test from 4 min > 5 sec.	172	43.3	3	5.8
Gait				
Two or more falls in the last year	122	30.7	4	7.7
Walking difficulties preventing to perform some daily activity	109	27.5	1	1.9
Sphincteral incontinence: involuntary loss of urine or feces	189	47.6	9	17.3
Communication				
Vision problems that may prevent the performance of some daily activity^d^	75	18.9	1	1.9
Hearing problems that may prevent the performance of some daily activity^e^	63	15.9	1	1.9
Multiple comorbidities: five or more chronic diseases or daily use of five or more different drugs or hospitalization in the last six months	153	38.5	1	1.9

ADL: activities of daily living; CRI: Reference Center for Older Adults; BMI: body mass index

^a^ Proportions estimated in relation to patients whose relative/friend have mentioned forgetfulness.

^b^ Examples of small domestic chores, mentioned in the questionnaire: washing dishes, cleaning the house, moderate housekeeping).

^c^ Positive for unintentional weight loss for individuals who, unintentionally: had lost more than 4.5 kg or 5.0% of body weight in the last year or 3 kg in the last month or 6 kg in the last six months.

^d^ Use of glasses/contact lenses allowed.

^e^ Use of hearing aids allowed.

The comparison of both groups indicated differences in the proportions (p < 0.001). The group attended at the CRI was older and more vulnerable to all dimensions of clinical-functional vulnerability. Regarding final average scores, standard deviations (SD), and median scores, respectively, they were equal to 12.6; 8.8; and 11 for older adults attended at CRI; and 1.98; 4.5; and 1 for older adults attended at the community.

### Association between IVCF-20 and CGA

Spearman’s correlation between the IVCF-20 and the CGA for the sample of older adults attended at the CRI was 0.790 (p < 0.001), indicating a correlation of high and positive magnitude; this value was 0.305 (p = 0.026) between older adults attended at the health center, indicating a positive correlation.

### Validity

The AUC-ROC statistics was equal to 0.903 (95%CI 0.871–0.934), being significantly higher than 0.50 and very close to 1 (Figure). The cut-off point obtained was 6 (six) and, therefore, values higher than six indicate frail older adults. For this value, the percentage classified as correct (accuracy) was 84.4% (335/397), this being the highest accuracy among all the possible cut-off points obtained. Thus, we identified the following values of sensitivity, specificity, positive predictive value, and negative predictive value, respectively: 90.5% (247/273); 71.0% (88/124); 87.3% (247/283); and 77.2% (88/114).

**Figure:**
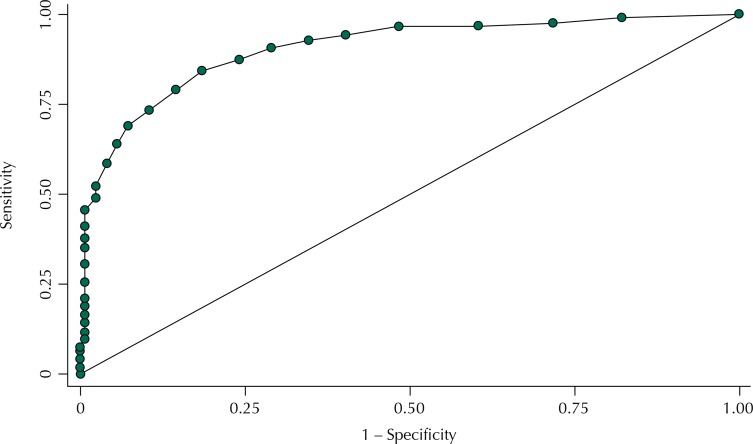
ROC curve evaluating sensitivity and specificity for different cut-off point values.

Area under the ROC curve = 0.9025

IVCF-20: Clinical-Functional Vulnerability Index-20; ROC: *Receiver Operating Characteristic*

### Reliability

Regarding the evaluators, evaluator A, in a higher proportion than evaluator B, indicated forgetfulness of the patient mentioned by a relative or friend, and dismay, sadness or hopelessness; compared with evaluator A, evaluator B mentioned higher proportion of self-perception of regular or bad health, worsening of forgetfulness, loss of interest in activities previously considered enjoyable, loss of aerobic/muscle capacity, sphincteral incontinence, vision problems, and comorbidities. Still, the averages and medians of the evaluators were very close and indicated values below three (older adult classified as robust). The averages of the evaluators were 1.7 (SD = 3.9) and 2.0 (SD = 4.4); and the median value for both was equal to 1.

The percentages of agreement and kappa coefficients were high. Kappa coefficients were almost perfect or substantial to all variables constituent of the IVCF-20, except for the variable “dismay, sadness, or hopelessness in the last month”, which obtained moderate agreement (Table 2). The quadratic weighted agreement was 99.5% for the IVCF-20 global index. Regarding the quadratic weighted kappa for the IVCF-20 global index, it was equal to 0.94, being considered, thus, almost perfect.

**Table 2:** Frequencies (absolute and percentage values) of clinical-functional vulnerability indicators regarding the studied population – distinct interviewers in both samples with the same individuals.

Clinical-Functional Vulnerability Indicators	Evaluator A		Evaluator B		Agreement percentage	Kappa coefficient
	
	n	%	n	%		
Regular or bad self-perception of health	5	9.6	10	19.2	90.4	0.62
Functional disabilities – instrumental ADLs						
Stopped, for health or physical condition...						
doing shopping	1	1.9	1	1.9	100	1.00
controlling money/expenditures/paying bills	1	1.9	1	1.9	100	1.00
doing small domestic chores	1	1.9	1	1.9	100	1.00
Basic ADL: do not bath alone	1	1.9	1	1.9	100	1.00
Cognition						
A relative or friend mentioned forgetfulness	9	17.3	5	9.6	92.3	0.67
Worsening of forgetfulness in the last months^b^	1	11.1^a^	2	40.0^a^	98.1	0.66
Forgetfulness preventing the performance of some daily activity^b^	0	0^a^	0	0^a^	100	NE
Mood						
Dismay/Sadness/Hopelessness in the last month	5	9.6	2	3.9	94.2	0.55
Loss of interest or pleasure, in the last month, in previously enjoyable activities	1	1.9	2	3.9	98.1	0.66
Mobility: reach, graspingness, and pincer grip						
Inability to raise the arm above shoulder level	0	0	0	0	100	NE
Inability to handle or hold small objects	0	0		0	100	NE
Aerobic and muscle capacity: Unintentional weight loss^c^ or BMI < 22 Kg/m^2^ or calf circumference < 31 cm or time during the gait speed test from 4 min > 5 sec.	2	3.9	3	5.8	98.1	0.79
Gait						
Two or more falls in the last year	4	7.7	4	7.7	100	1.00
Walking difficulties preventing to perform some daily activity	1	1.9	1	1.9	100	1.00
Sphincteral incontinence: involuntary loss of urine or feces	8	15.4	9	17.3	94.2	0.79
Communication						
Vision problems that may prevent the performance of some daily activity^d^	0	0	1	1.9	98.1	NE
Hearing problems that may prevent the performance of some daily activity^e^	1	1.9	1	1.9	100	1.00
Comorbidities: Five or more chronic diseases or daily use of five or more different drugs or hospitalization in the last six months	0	0	1	1.9	98.1	NE

ADL: activities of daily living; NE: not estimated; BMI: body mass index

^a^ Proportions estimated in relation to patients whose relative/friend have mentioned forgetfulness.

^b^ Examples of small domestic chores, mentioned in the questionnaire: washing dishes, cleaning the house, moderate housekeeping.

^c^ Positive for unintentional weight loss for individuals who, unintentionally: had lost more than 4.5 kg or 5.0% of body weight in the last year or 3 kg in the last month or 6 kg in the last six months.

^d^ Use of glasses/contact lenses allowed.

^e^ Use of hearing aids allowed.

Cronbach’s alpha coefficients found to older adults attended at the CRI and at the health center were, respectively, 0.740 (value considered reputable) and 0.861 (value considered very good).

## DISCUSSION

This study compared IVCF-20 with CGA. Firstly, we evaluated the correlation between the results obtained from both evaluations, to verify if they had a relation, which was done by Spearman’s rankings correlation. Thus, we could confirm that high values obtained by an instrument were also high in another instrument. This result was expected, considering that the evaluations have similar objectives to classify older adults regarding fragility. In addition, it was obtained in a less frail population and in another more frail population, which confirms the feasibility of IVCF-20 application in distinct populations.

After analyzing the existence of a positive relationship between both variables, the validity of the IVCF-20 instrument was checked by the AUC-ROC statistics. Such checking was made for older adults attended at the CRI and the instrument was considered valid. We also obtained the cut-off point to discriminate frail older adults based on the criterion of higher accuracy. Based on this criterion, we obtained tests with sensitivity higher than 90.0% and specificity higher than 70.0%. This result of high sensitivity, even to the detriment of higher specificity, is desirable, considering that triage tests (screening) should have high sensitivity to not miss sick individuals (false negatives)^9^.

Regarding reliability, we deliberately made an effort to assess this reliability among both older adults at the health center and those at the CRI. All the indicators measured in the health center indicated that the instrument was reliable: high agreement statistics and high kappa coefficient as well as Cronbach’s alpha coefficient. In the case of the CRI, the alpha coefficient was the only evaluated, but followed the results obtained at the health center.

The findings concerning the kappa statistic indicated stability between the evaluators, a fundamental condition in defining the instrument. On the other hand, the results of the Cronbach’s alpha statistics found in this study indicate that the questions that compose the IVCF-20 measure the same construct (alpha > 0.70) and that the questions are not redundant (alpha < 0.90). Hence, this is an instrument considered reliable in the population evaluated.

It is an instrument easy to use and of fast application. Therefore, the IVCF-20 proved to be a good instrument for the initial identification of older adults at risk, able to recognize the older adult that needs be subjected to an assessment performed by a specialized geriatric/gerontological team.

Thus, IVCF-20 can be considered a CGA methodology performed by professionals who are non-specialists in geriatrics and gerontology, and can be applied by middle-level professionals previously trained. However, it should be noted that this is an initial triage instrument. Other possible applications of the instrument would be:

- Indication of interdisciplinary interventions able to improve the autonomy and independence of the older adult and preventing functional decline, institutionalization and death, for those older adults to whom it was impossible to apply CGA. Even though the CGA application to all older adults is vital, few regions of Brazil have specialists in geriatrics and gerontology. In this situation, IVCF-20 may suggest several preventive measures that may be useful for older adults and their family until the specialized geriatric/gerontological evaluation is possible;
- Managerial function, as a qualifier instrument of vulnerability, allowing identification and monitoring of the population at most risk for hospitalization and overuse of health equipment;
- Schedule demand planning on primary health care such as the definition of a group of older adults who will require a unique care in primary health care;
- Structuring and guidance of specialized geriatric/gerontological consultation: planning of the specialized consultation of older adults, highlighting the dimensions of their health that deserve a more detailed investigation.

The application of the instrument must also be diversified for other modalities of care to older adults, such as geriatric clinics, community centers, emergency services, and long-stay institutions.

This study has limitations. Due to the fact that it included older adults from just one community health center and a center of reference under geriatric secondary care, it may not be representative of the target – that is, the older adults population. In addition, it is a convenience sample, and it is possible that frailer older adults are not represented. Thus, the elderly of this study may be healthier than the overall population of older adults. Additionally, although the number of patients obtained is considerable (over 400 older adults), the results have limited capacity to be extrapolated to other municipalities or other regions of Minas Gerais.

In conclusion, this instrument can be used for initial triage in primary health care. However, it is worth noting that the IVCF-20 does not replace the evaluation performed by the specialized geriatric-gerontological team. The frail older adult needs a specialized approach, in a reference unit for older adults, and a complete multidimensional assessment is essential for a correct therapeutic interdisciplinary project.

## Footnotes

a Clinical-Functional Vulnerability Index (IVCF-20). Full questionnaire available at: www.ivcf20.com.br
